# Effects of Melatonin on Glucose Homeostasis, Antioxidant Ability, and Adipokine Secretion in ICR Mice with NA/STZ-Induced Hyperglycemia

**DOI:** 10.3390/nu9111187

**Published:** 2017-10-29

**Authors:** Chung-Cheng Lo, Shyh-Hsiang Lin, Jung-Su Chang, Yi-Wen Chien

**Affiliations:** 1 Department of Nutrition and Health Sciences, Taipei Medical University, Taipei 11031, Taiwan; b506100016@tmu.edu.tw (C.-C.L.); lin5611@tmu.edu.tw (S.-H.L.); susanchang@tmu.edu.tw (J.-S.C.); 2 Research Center of Geriatric Nutrition, College of Nutrition, Taipei Medical University, Taipei 11031, Taiwan

**Keywords:** melatonin, diabetes, adiopkines, oxidative stress and insulin resistance

## Abstract

Diabetes is often associated with decreased melatonin level. The aim was to investigate the effects of different dosage of melatonin on glucose hemostasis, antioxidant ability and adipokines secretion in diabetic institute for cancer research (ICR) mice. Forty animals were randomly divided into five groups including control (C), diabetic (D), low-dosage (L), medium-dosage (M), and high-dosage (H) groups. Groups L, M, and H, respectively, received oral melatonin at 10, 20, and 50 mg/kg of BW (body weight) daily after inducing hyperglycemia by nicotinamide (NA)/ streptozotocin (STZ). After the six-week intervention, results showed that melatonin administration increased insulin level and performed lower area under the curve (AUC) in H group (*p* < 0.05). Melatonin could lower hepatic Malondialdehyde (MDA) level in all melatonin-treated groups and increase superoxide dismutase activity in H group (*p* < 0.05). Melatonin-treated groups revealed significant higher adiponectin in L group, and lower leptin/adiponectin ratio and leptin in M and H groups (*p* < 0.05). Melatonin could lower cholesterol and triglyceride in liver and decrease plasma cholesterol and low-density lipoprotein-cholesterol (LDL-C) in L group, and increase plasma high-density lipoprotein-cholesterol (HDL-C) in H group (*p* < 0.05). Above all, melatonin could decrease oxidative stress, increase the adiponectin level and improve dyslipidemia, especially in H group. These data support melatonin possibly being a helpful aid for treating hyperglycemia-related symptoms.

## 1. Introduction

The prevalence of type 2 diabetes mellitus (DM) has increased over short periods and occurs at a relatively younger age and lower body-mass index in Asia [1]. When people in Asia develop diabetes with a lower degree of obesity at younger ages, they suffer longer from diabetic complications and die sooner than people in other regions [2]. In Taiwan, DM was the fifth leading cause of death in 2015, and its prevalence has been increasing [3]. Complications of DM include macrovascular and microvascular complications such as cardiovascular disease, chronic kidney failure, and diabetic retinopathy [4,5,6]. Those complications are attributed to hyperglycemia inducing oxidative stress. Hyperglycemia increases NADH and FADH_2_ and inhibits the delivery of protons through complex III in electron transport chain, which leads to overproduction of reactive oxygen species (ROS) and oxidative stress [7]. ROS are highly toxic to cell components, particularly to cell membranes composed of lipids. ROS destroy lipids on membranes and produce lipid peroxide which damages cells. ROS are metabolized by nonenzymatic and enzymatic mechanisms to eliminate oxidative stress [8].

Melatonin (5-methoxy-*N*-acetyltryptamine), which is one of the strongest antioxidants, is secreted with a daily rhythm by the pineal gland [9]. The peak concentration is around 10 pg/mL (43 pmol/L) in blood and 3 pg/mL in the saliva [10]. It is thought that melatonin may be useful in the management of several diseases, such as depression [11], insomnia [11,12], obesity [9,13], diabetes [13], cancer [12], and immune [14] and cardiac disorders [15]. It can scavenge ROS by its antioxidant properties and increase antioxidant enzyme activities [16]. A recent study indicated that animals which received a pinealectomy exhibited impaired glucose tolerance, insulin resistance, and diabetes, which were improved by treatment with melatonin [17]. In high-cholesterol diet rat model, supplementation with melatonin could decrease plasma total cholesterol and liver cholesterol and triglyceride, and increase plasma high-density lipoprotein-cholesterol (HDL-C) [18]. In Zucker diabetic fatty (ZDF) rats, supplementation with melatonin not only decreased elevated plasma leptin, insulin and high blood glucose, but increased low levels of adiponectin [13]. In a rat model of streptozotocin (STZ)-induced DM, supplementation with melatonin decreased fasting blood glucose (FBG) and improved liver damage, and reduced the oxidative stress [19].

To induce diabetes, it is common to use STZ in a rodent model. STZ, an antibiotic produced by *Streptomyces achromogens*, is a 2-deoxy-d-glucose derivative, and because its structure is like that of glucose, it can cross glucose transporter 2 on islets [20]. The damage to pancreatic cells by STZ was proposed to occur by two mechanisms. One is that STZ is a strong alkylating agent, which directly alkylates DNA by •CH3 or CH_3_^+^ when STZ decomposes. The other is that STZ generates ROS in diabetogenesis [21]. However, Murata et al. indicated that diabetogenesis of STZ is mainly caused by alkylation of DNA by CH_3_^+^ [22]. A method of inducing type 2 DM with hyperglycemia and relatively low insulin levels can be produced by combining STZ and nicotinamide (NA) [23,24]. In an ICR mice model, the combination of STZ and NA resulted in moderate hyperglycemia and metabolic syndrome [25].

The intervention with melatonin can improve FBG, insulin, adipokines, and the oxidative state in a DM rat model [13,19]. However, few studies have evaluated the intervention with melatonin in a DM mouse model, and how melatonin affects glucose levels in an STZ-induced diabetic model is still controversial. These experiments were carried out to determine the effects of different doses of melatonin on glucose tolerance, lipid profile, adipokines, and activities of antioxidant enzymes in mice with NA/STZ-induced hyperglycemia.

## 2. Materials and Methods

### 2.1. Chemicals

STZ and melatonin were purchased from Sigma Chemical (St. Louis, MO, USA).

### 2.2. Animals

Male ICR mice (28–30 g body weight (BW)) were obtained at an age of 8 weeks from BioLASCO Taiwan (Taipei, Taiwan). Mice were maintained on commercial standard chow and tap water ad libitum. Mice were housed in a temperature- (22 ± 1 °C) and humidity- (40–60%) controlled room with a 12-h dark/light cycle (lights on at 07:00). BWs were recorded weekly throughout the experiments. After 1 week of acclimatization, animals were randomly divided into five groups including control (C), diabetic (D), low-dosage (L), medium-dosage (M), and high-dosage (H) groups. Groups C and D received the vehicle (a 6% alcohol (*w*/*v*) aqueous solution) daily. Groups L, M, and H, respectively, received melatonin dissolved in a 6% alcohol solution at 10, 20, and 50 mg/kg of BW daily after inducing hyperglycemia immediately. The vehicle and melatonin solution were given by oral tube feeding between 17:00 and 19:00 daily for 6 weeks. At the end of the experiment, mice were sacrificed by anesthetization. In addition, six normal male ICR mice (35–45 g BW) were used to study the pharmacokinetics of melatonin. This study was approved by the Institutional Animal Care and Use Committee (IACUC) of the Institute for Experimental Medical Research, Taipei Medical University (Taipei, Taiwan; LAC-2016-0173).

### 2.3. Induction of Hyperglycemia

The D, L, M and H groups were intraperitoneally (i.p.) injected with STZ (50 mg/kg of BW) in 0.1 M citrate buffer (pH 4.2) on two consecutive days. NA (120 mg/kg of BW) in saline was i.p. injected 30 min before the STZ injection on the first day after overnight fasting. Group C was i.p. injected with citrate buffer. Seven days after the second i.p. injection, mice that exhibited an 8-h FBG level of ≥200 mg/dL were recognized as being hyperglycemic. The others that exhibited an FBG level of <200 mg/dL were injected with STZ and monitored until the FBG level reached ≥200 mg/dL. FBG was monitored with a glucometer (Dragon Pharmaceutical Co, New Taipei, Taiwan). After D, L, M and H groups were induced hyperglycemia, melatonin administration was carried out at the same time.

### 2.4. Oral Glucose Tolerance Test (OGTT)

The OGTT was performed at 09:00, and 12-h FBG of mice was monitored at 0 min. The glucose load (2 g/kg of BW) was given as a bolus by gavage, and blood sugar was monitored at 30, 60, 90, and 120 min after glucose administration.

### 2.5. Pharmacokinetics of Melatonin

Six mice were starved for 4 h before being given melatonin by gavage. Melatonin was prepared as previously described. On Day 1, mice were tube fed melatonin at a dosage of 10 mg/kg of BW. Blood samples were collected from a tail vein at 10, 30, 60, and 120 min after melatonin administration. Blood samples were centrifuged at 1000× *g* for 30 min, and supernatants were collected to determine the melatonin concentration. On Day 5, the same six mice were administered melatonin at 50 mg/kg of BW and then followed the same steps as Day 1. The elimination rate constant (Ke) was calculated by (ln(Cp1)–ln(Cp2))/(t_2_–t_1_), where Cp1 is the melatonin concentration at t_1_, and Cp2 is the melatonin concentration at t_2_. The half-life (t_1/2_) was calculated by Ln(2)/Ke.

### 2.6. Biochemical Analysis

Plasma insulin was determined by a Mercodia mouse insulin enzyme-linked immunosorbent assay (ELISA) kit (Uppsala, Sweden). Plasma leptin and adiponectin were determined by an AssayMax mouse leptin and adiponectin ELISA kit (St. Charles, IL, USA). Plasma and serum melatonin were determined by a mouse melatonin ELISA kit (MyBioSource, Inc., San Diego, CA, USA). Plasma and liver total cholesterol and triglyceride were determined by a colorimetric assay kit. The homeostasis model assessment for insulin resistance (HOMA-IR) and quantitative insulin sensitivity check index (QUICKI) were calculated using the fasting glucose level (mg/dL) and insulin concentration (µU/mL). The HOMA-IR was calculated by (fasting blood sugar × insulin)/405. QUICKI was calculated by 1/(log(fasting blood sugar) + log(insulin)). Malondialdehyde (MDA) in liver homogenates and plasma was measured by a thiobarbituric acid-reactive substance (TBARS) assay kit from Cayman Chemical (Ann Arbor, MI, USA). Activities of superoxide dismutase (SOD) and glutathione peroxidase (GPx) in liver homogenates were measured by kits from Cayman Chemical. Total protein concentrations were determined by Bio-Rad protein assay (Bio-Rad Laboratories, Inc., Hercules, CA, USA).

### 2.7. Statistical Analysis

Data were analyzed using PASW Statistics software (vers. 18.0, SPSS, Chicago, IL, USA). Comparisons between groups were made with an analysis of variance (ANOVA) followed by Duncan’s multiple-range test. A *p* value of <0.05 was considered significant.

## 3. Results

### 3.1. Body, Fat Mass, and Organ Weights

Table 1 shows no significant differences between groups in body weight during the experimental period. Groups L, M, and H were significantly thinner than Group C, and Group H was significantly thinner than Group D. However, absolute and relative epididymal fat mass weights in Group C were greater than those in the D and melatonin-treated groups, and there was no significant difference between Group D and treated groups (Groups L, M, and H). No significant differences in absolute or relative kidney weights were observed between groups. Absolute and relative liver weights in Group D were significantly heavier than those in Group C, but, after the intervention, only those of Group M were significantly lighter than those of Group D.

### 3.2. Water Intake

Table 2 shows that throughout the experimental period, water intake in Group D and treated groups (Groups L, M, and H) was significantly higher than that in Group C. At Week 2, Group H drank more water than Group D. After the intervention, although the treated group did not exhibit a reduction in water intake, Group D had the greatest increase in water intake, which was significantly greater than that of the treated group.

### 3.3. Fasting Glucose, Plasma Insulin, and HOMA-IR

Table 3 shows that, at Week 0, no significant difference in the 12-h FBG level was observed. At Week 6, significant increases in 12-h FBG levels were found in Group D and treated groups (Groups L, M, and H) compared to Group C. This NA/STZ-induced model performed high blood glucose, relative low plasma insulin level and normal body weight in Group D, which might be more like to be type 2 DM. Compared to the increased glucose level from Week 2 to Week 6, the greatest increase in glucose levels was observed in Group D and was significantly higher than those of groups C, M, and H. Plasma insulin in Group D was significantly lower than that in Group C; Group H had a significantly higher insulin level than Group D. After calculating the FBG and plasma insulin, the HOMA-IR were calculated. The HOMA-IR were significantly higher in Group D compared to those of Group C, but melatonin did not improve the HOMA-IR in the melatonin-treated groups.

### 3.4. Glucose Homeostasis

**Figure 1:**
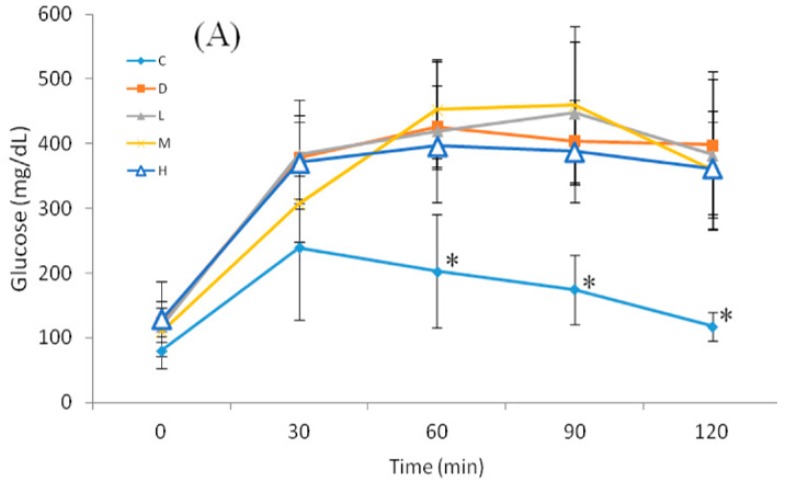

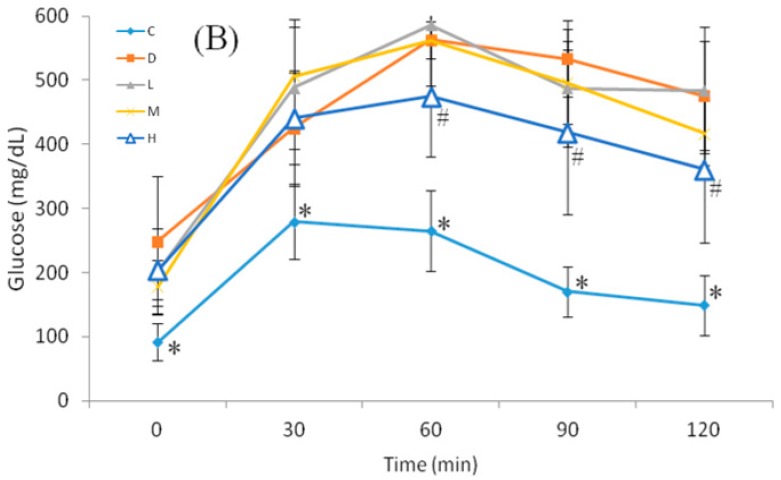
Oral glucose tolerance test (OGTT): (**A**) before; and (**B**) after the six-week melatonin intervention. All values are the mean ± SD (*n* = 8). * *p* < 0.05 compared to the diabetic group. # *p* < 0.05 compared to the diabetic group. C, control group; D, diabetic group; L, low-dosage group (10 mg melatonin/kg of body weight (BW)); M, medium-dosage group (20 mg melatonin/kg of BW); and H, high-dosage group (50 mg melatonin/kg of BW).

Values recorded for the Oral glucose tolerance test (OGTT) are presented in Figure 1. At Week 0, there was no significant difference in blood glucose levels at 0 min. At 60 min after feeding glucose, the D and treated groups had significantly higher blood glucose than Group C, and this continued to 120 min. Values recorded for drawing of the AUC are presented in Table 4. The AUCs in the D and treated groups were significantly higher than that in Group C, and no significant difference between the D and treated groups was observed.

At Week 6, significantly higher blood glucose levels in Group D and treated groups (Groups L, M, and H) were observed compared to Group C at 0–120 min. Nevertheless, it was noted that blood glucose levels in Group H were significantly lower than those Group D at 60–120 min. The AUCs in Group D and treated groups (Groups L, M, and H) were significantly higher than that in Group C, but it was observed that the AUC in Group H was significantly lower than that in Group D.

### 3.5. Liver and Plasma Lipid Profiles

As shown in Table 5, no significant difference between groups C and D in hepatic triglyceride was observed. Hepatic cholesterol in Group D was significantly higher than that in Group C. Liver cholesterol and triglyceride levels in all treated groups were significantly lower compared to those in Group D. Plasma triglyceride, cholesterol, and low-density lipoprotein-cholesterol (LDL-C) in Group D were significantly higher than those in Group C, but plasma HDL-C did not differ from that in Group C. It was observed that plasma cholesterol, LDL-C and total cholesterol/HDL-C ratio were significantly lower in Group L. Plasma triglyceride, LDL-C and total cholesterol/HDL-C ratio were significantly lower, and plasma HDL-C was significantly higher in Group H compared to Group L. It might indicate that high dosage melatonin exerted better improvement of blood lipid composition than low dosage melatonin.

### 3.6. Oxidative Stress Biomarkers

As shown in Table 6, liver and plasma MDA levels in Group D were significantly higher and liver SOD and GPx activities were significantly lower than those in Group C. Although supplementation with melatonin failed to increase GPx activity, SOD activity in Group H was significantly higher and MDA in groups M and H were significantly lower compared to those in Group D.

### 3.7. Plasma Adipokines and Melatonin

Values recorded for adipokines and melatonin are presented in Table 7. Plasma adiponectin levels in Group D were significantly lower than those in Group C, but the plasma leptin level and leptin/adiponectin ratio did not differ from those of Group C. All of the melatonin-treated groups had significantly higher plasma adiponectin levels than Group D, but they were still lower compared to those of Group C. Groups M and H had significantly lower plasma leptin levels than those of groups C and D. The leptin/adiponectin ratio in Group L was lower than that in Group D, and those in groups M and H were lower than that in Group L. Plasma melatonin levels in Group D were significantly lower than those in Group C. The melatonin-treated groups had significantly higher plasma melatonin levels compared to Group D. Group L had significantly higher plasma melatonin levels compared to those of Group C, and those of groups M and H were significantly higher than that of Group L.

### 3.8. Pharmacokinetics of Melatonin and Plasma Melatonin

As shown in Table 8, the 50-mg/kg of BW group had significantly higher serum melatonin concentrations at 10, 30, 60, and 120 min compared to the 10-mg/kg of BW group. The AUC in the 50-mg/kg of BW group was significantly larger than that in the 10-mg/kg of BW group. However, there was no significant difference in the half life and Ke between the 10- and 50-mg/kg BW groups.

## 4. Discussion

Melatonin is a biological modulator of mood, sleep, sexual behavior and circadian rhythm at physiological concentration in human [16]. However, melatonin secretion decreases with age along with increased insulin resistance, but supplement of melatonin can relieve those symptoms [16]. In a published cell experiment, 10 nM melatonin increased the ability of glucose intake in 3T3-L1 adipocytes [26]. In this study, administration of melatonin at 50 mg/kg of BW increased and maintained serum melatonin levels ranging 1.46–11.67 µM, which was 1000 times than nM. It might indicate that administration of melatonin at 50 mg/kg can reach effective concentration of melatonin. After STZ induction of diabetes, rats exhibited high blood glucose and low plasma insulin levels. Moreover, those rats also exhibited low melatonin levels [27]. This study showed that Group D exhibited low plasma melatonin levels and high glucose levels after NA/STZ induction, and an oral melatonin intervention recovered plasma melatonin levels. According to previous studies, melatonin administration can reduce BW, fat mass, FBG, and insulin levels in gene- or diet-induced diabetic [13,28,29,30,31,32] obese rodents. Nevertheless, it is still controversial that the effects of melatonin on blood glucose in an STZ-induced diabetic model. Several researchers reported that supplementation with melatonin did not lower FBG in an STZ diabetic rat model [33,34,35,36]. In contrast, other researchers reported that 10–20 mg/kg melatonin exerted lower FBG in an STZ- or alloxan-induced diabetic rat model [37,38,39]. The current study showed that oral melatonin administration did not improve FBG and HOMA-IR in mice with NA/STZ-induced hyperglycemia, which corresponded to the latter results for FBG. However, Group H showed a significantly lower AUC compared to Group D. This may indicate that melatonin delayed diabetic progression by increasing glucose sensitivity and the insulin level.

Hyperglycemia usually results in hypertriglyceridemia and hypercholesterolemia [40]. STZ is able to induce hyperglycemia and results in hypertriglyceridemia and hypercholesterolemia [25]. In this study, plasma triglyceride and cholesterol and the hepatic cholesterol level in Group D significantly increased. In previous studies, melatonin administration lowered plasma triglyceride and cholesterol and increased plasma HDL-C level at 10–20 mg/kg in a gene- [41] or diet-induced [28,29,31] obese rat model and an STZ-induced diabetic rat model [38,39]. In high-cholesterol diet rat model, melatonin could decrease plasma total cholesterol and liver cholesterol and triglyceride, and increase plasma HDL-C, which exerted similar effect as hypocholesterolemia agents, cholestyramine [18]. The current study showed that oral melatonin administration could increase plasma HDL-C level and lower triglyceride and cholesterol in both the plasma and liver of mice with NA/STZ-induced diabetes, which corresponded to previous studies. Melatonin could increase HDL-C level along with lower plasma cholesterol, which leading to lower total cholesterol/HDL-C ratio. In previous study, plasma total cholesterol/HDL-C ratio the strongest independent predictors of development for atherosclerosis [42]. This may indicate that melatonin improve blood lipid composition, which is beneficial to diabetes patients.

In diabetic models, it is usually observed that oxidative stress increases. Several studies reported that increased MDA levels in diabetic models were decreased by melatonin, regardless of whether the glucose level was lower or not [19,30,34,37,43,44]. In this study, melatonin lowered MDA levels in the liver and plasma. However, SOD and GPx activities may increase [19,37,44,45], decrease [44,46] or remain unchanged [43,46] in diabetic models. In some research, melatonin did not increase SOD or GPx activities [43,46]. However, other researchers reported that melatonin increased SOD or GPx activities [35,37,44]. In this study, both hepatic SOD and GPx activities were lower in Group D. Melatonin administration increased liver SOD activity but not GPx activity. Nevertheless, melatonin decreased MDA levels and increased melatonin levels in all intervention groups, but did not significantly increase SOD activities in groups L or M. Otherwise, because melatonin’s strong anti-oxidative ability, increased plasma melatonin levels in all intervention groups might also exert higher anti-oxidative ability. It was assumed that melatonin mainly reduced MDA levels by its anti-oxidative property in groups L and M, and reduced MDA levels by combining its anti-oxidative property with increasing SOD activity in Group H.

In a gene-induced obesity diabetic model, leptin secretion increased along with increasing fat, but adiponectin secretion decreased, leading to an increase in the leptin/adiponectin ratio. However, supplementation with melatonin can increase adiponectin and decrease leptin levels in this model [13]. Moreover, supplementation with melatonin can decrease leptin secretion and increase adiponectin secretion in a diet-induced model [28,30,31]. On the other hand, leptin and adiponectin decreased in an NA/STZ-induced rat model, and adipocytes exerted lower glucose intake and oxidation ability [23,24]. Few studies reported effects of melatonin on adipokines in an NA/STZ-induced diabetic model. De Oliveira et al. reported that supplementation with melatonin can increase adiponectin and lower leptin levels in a neonatal STZ-induced diabetic model [47]. In this study, melatonin increased adiponectin in all intervention groups and lowered leptin levels in groups M and H, which corresponded to results of de Oliveira and coworkers’ study. The leptin/adiponectin ratio is considered a better anti-atherogenic index than adiponectin alone [48]. In our study, melatonin lowered the leptin/adiponectin ratio in Group L, as it did in groups M and H compared to Group L.

Although oral administration of melatonin did not decrease blood glucose, it improved hyperglycemia-induced high oxidative stress, low adiponectin levels, and dyslipidemia. Otherwise, melatonin could improve blood lipid composition and decrease total cholesterol/HDL-c ratio, which was thought to reduce the risk atherogenesis. If diabetic patients receive an oral hypoglycemic agent with melatonin administration to control blood glucose, it might be beneficial to improve diabetes-related phenomena.

## 5. Conclusions

In conclusion, in this study, six-week oral melatonin administration at 10 mg/kg of BW was able to decrease plasma and liver cholesterol and plasma MDA levels, and increase the adiponectin level. Oral melatonin administration at 50 mg/kg of BW further reduced the leptin/adiponectin ratio and plasma triglyceride, delayed the blood glucose increase during intervention period, and improved glucose tolerance. These experimental data contribute to supporting melatonin possibly being a helpful aid for treating diabetes mellitus and metabolic syndrome, as it may exert an anti-atherogenic effect. Further research is needed in clinical trials to assess whether melatonin administration in humans would be similarly beneficial

## Figures and Tables

**Table 1:** Body, fat mass, and organ weights after the six-week melatonin intervention.

Group	C	D	L	M	H
Fasting body weight (g)	36.47 ± 1.65 ^a^	36.18 ± 1.01 ^a,b^	34.47 ± 1.79 ^b,c^	35.35 ± 1.44 ^b,c^	33.80 ± 2.24 ^c^
Initial body weight (g)	37.28 ± 2.09	39.12 ± 1.91	37.41 ± 2.08	39.27 ± 1.19	37.28 ± 2.43
Final body weight (g)	38.83 ± 1.55	40.71 ± 1.38	38.28 ± 1.66	39.80 ± 2.06	38.70 ± 2.66
Total weight gain (g)	1.15 ± 2.07	1.65 ± 1.70	1.06 ± 0.36	0.61 ± 0.91	1.31 ± 0.18
Liver weight (g)	1.75 ± 0.18 ^a^	2.06 ± 0.24 ^b^	1.90 ± 0.13 ^a,b^	1.84 ± 0.10 ^a^	1.92 ± 0.18 ^a,b^
Kidney weight (g)	0.66 ± 0.08	0.65 ± 0.09	0.63 ± 0.08	0.62 ± 0.05	0.62 ± 0.07
Epididymal fat mass weight (g)	0.52 ± 0.13 ^a^	0.32 ± 0.21 ^b^	0.34 ± 0.11 ^b^	0.32 ± 0.07 ^b^	0.24 ± 0.08 ^b^
Relative liver weight (g/100 g BW)	4.79 ± 0.34 ^a^	5.70 ± 0.56 ^c^	5.54 ± 0.47 ^b,c^	5.22 ± 0.25 ^a,b^	5.69 ± 0.34 ^c^
Relative kidney weight (g/100 g BW)	1.81 ± 0.18	1.79 ± 0.20	1.83 ± 0.19	1.78 ± 0.12	1.84 ± 0.15
Relative epididymal fat mass weight (g/100 g BW)	1.42 ± 0.37 ^a^	0.91 ± 0.58 ^b^	1.00 ± 0.32 ^b^	0.92 ± 0.19 ^b^	0.71 ± 0.25 ^b^

All value are the mean ± SD (*n* = 8); ^a, b, c^ Values with different superscripts significantly differ between groups (*p* < 0.05); C, control group; D, diabetic group; L, low-dosage group (10 mg melatonin/kg of body weight (BW)); M, medium-dosage group (20 mg melatonin/kg of BW); and H, high-dosage group (50 mg melatonin/kg of BW).

**Table 2:** Water intake during melatonin intervention at 2, 4, and 6 weeks.

Group	C	D	L	M	H
Water intake (mL/day)					
Week 2	7.70 ± 0.89 ^a^	18.89 ± 0.52 ^b^	20.95 ± 1.86 ^b^	18.86 ± 1.41 ^b^	23.97 ± 3.82 ^c^
Week 4	6.95 ± 0.49 ^a^	22.04 ± 1.50 *^,b^	23.33 ± 1.25 *^,b,c^	21.88 ± 0.79 *^,b^	24.41 ± 1.21 ^c^
Week 6	6.38 ± 0.37 *^,a^	26.06 ± 4.19 *^, b^	24.30 ± 3.02 *^,b^	22.38 ± 2.32 *^,b^	25.01 ± 3.69 ^b^
Total water gain (mL/day)	−1.04 ± 0.04 ^a^	7.18 ± 14.10 ^c^	3.34 ± 2.95 ^b^	3.52 ± 1.55 ^b^	1.03 ± 7.4 ^a,b^

All values are the mean ± SD (*n* = 8); ^a, b, c^ Values with different superscripts significantly differ between groups (*p* < 0.05); * Significant difference compared to Week 2 (*p* < 0.05); C, control group; D, diabetic group; L, low-dosage group (10 mg melatonin/kg of body weight (BW)); M, medium-dosage group (20 mg melatonin/kg of BW); and H, high-dosage group (50 mg melatonin/kg of BW).

**Table 3:** Fasting glucose, plasma insulin, homeostasis model assessment for insulin resistance (HOMA-IR) after the six-week melatonin intervention.

Group	C	D	L	M	H
Fasting glucose (mg/dL)					
Week 0	80.6 ± 27.6	124.6 ± 22.5	115.7 ± 37.2	109.6 ± 15.9	115.5 ± 67.3
Week 6	91.5 ± 29.2 ^a^	249.1 ± 101.1 ^b^	214.8 ± 80.8 ^b^	178.1 ± 41.5 ^b^	203.5 ± 45.2 ^b^
Change in fasting glucose (mg/dL)	9.0 ± 25.2 ^a^	124.5 ± 42.2 ^b^	107.8 ± 68.8 ^b,c^	80.2 ± 50.8 ^c^	74.3 ± 32.1 ^c^
Plasma insulin (µU/mL )	9.21 ± 0.71 ^a^	8.45 ± 0.56 ^b^	8.23 ± 0.58 ^b^	8.48 ± 0.53 ^a,b^	9.43 ± 0.94 ^a^
HOMA-IR	2.04 ± 0.67 ^a^	5.33 ± 2.32 ^b^	4.40 ± 1.65 ^b^	3.7 ± 0.75 ^b^	4.60 ± 0.80 ^b^

All values are the mean ± SD (*n* = 8); ^a, b, c^ Values with different superscripts significantly differ between groups (*p* < 0.05); C, control group; D, diabetic group; L, low-dosage group (10 mg melatonin/kg of body weight (BW)); M, medium-dosage group (20 mg melatonin/kg of BW); and H, high-dosage group (50 mg melatonin/kg of BW).

**Table 4:** Glucose tolerance before and after the six-week melatonin intervention.

Group	C	D	L	M	H
AUC					
Week 0	21,474 ± 6855 ^a^	44,128 ± 5314 ^b^	45,042 ± 5681 ^b^	43,616 ± 6426 ^b^	42,042 ± 5558 ^b^
Week 6	25,040 ± 5530 ^a^	56,508 ± 6158 ^b^	57,173 ± 4865 ^b^	55,865 ± 5394 ^b^	48,500 ± 9433 ^c^
Increase of AUC values	3566 ± 4484 ^a^	13,036 ± 2892 ^b^	12,130 ± 2358 ^b^	12,249 ± 3540 ^b^	6457 ± 4309 ^a^

All values are the mean ± SD (*n* = 8). ^a, b, c^ Values with different superscripts significantly differ between groups (*p* < 0.05). C, control group; D, diabetic group; L, low-dosage group (10 mg melatonin/kg of body weight (BW)); M, medium-dosage group (20 mg melatonin/kg of BW); and H, high-dosage group (50 mg melatonin/kg of BW).

**Table 5:** Liver and plasma lipids after the six-week melatonin intervention.

Group	C	D	L	M	H
Liver					
Triglyceride (mg/g tissue)	18.5 ± 4.7 ^a^	16.4 ± 3.0 ^a^	10.4 ± 1.5 ^b^	10.0 ± 2.2 ^b^	9.3 ± 2.2 ^b^
Cholesterol (mg/g tissue )	3.24 ± 0.96 ^a^	5.69 ± 0.98 ^b^	3.22 ± 0.75 ^a^	3.16 ± 0.51 ^a^	3.14 ± 0.70 ^a^
Plasma					
Triglyceride (mg/dL)	78.0 ± 15.4 ^a^	114.2 ± 15.0 ^b^	103.1 ± 19.7 ^b^	95.8 ± 14.6 ^a,b^	81.5 ± 18.8 ^a^
Cholesterol (mg/dL)	74.4 ± 12.5 ^a^	116.4 ± 12.5 ^c^	94.5 ± 17.8 ^b^	91.3 ± 12.3 ^b^	90.8 ± 10.7 ^b^
HDL-C (mg/dL)	38.0 ± 11.2 ^a^	39.2 ± 9.1 ^a^	39.0 ± 6.1 ^a^	45.8 ± 13.0 ^a^	59.6 ± 7.6 ^b^
LDL-C (mg/dL)	22.0 ± 9.9 ^a,b^	54.4 ± 10.7 ^c^	35.0± 17.5 ^b^	28.9 ± 9.6 ^b^	14.9 ± 6.2 ^a^
Total Cholesterol/HDL-C ratio	2.12 ± 0.55 ^a^	3.53 ± 0.67 ^b^	2.48 ± 0.65 ^a^	2.08 ± 0.29 ^a^	1.53 ± 0.18 ^c^
LDL-C/HDL-C ratio	0.67 ± 0.43 ^a,c^	1.83 ± 0.56 ^b^	0.93 ± 0.54 ^a^	0.64 ± 0.25 ^a,c^	0.25 ± 0.12 ^c^

All values are the mean ± SD (*n* = 8). ^a, b, c^ Values with different superscripts significantly differ between groups (*p* < 0.05). C, control group; D, diabetic group; L, low-dosage group (10 mg melatonin/kg of body weight (BW)); M, medium-dosage group (20 mg melatonin/kg of BW); H, high-dosage group (50 mg melatonin/kg of BW); HDL-C, high-density lipoprotein-cholesterol; and LDL-C, low-density lipoprotein-cholesterol.

**Table 6:** Plasma and liver malondialdehyde (MDA), superoxide dismutase (SOD) and glutathione peroxidase (GPx) activity after the six-week melatonin intervention.

Group	C	D	L	M	H
Liver					
MDA (nmol/g tissue)	91.2 ± 16.1 ^a^	127.2 ± 10.7 ^b^	93.1 ± 18.5 ^a^	95.4 ± 14.7 ^a^	84.4 ± 22.3 ^a^
SOD (U/mg protein)	2.18 ± 0.96 ^a^	1.12 ± 0.11 ^b^	1.43 ± 0.30 ^b,c^	1.52 ± 0.47 ^b,c^	1.75 ± 0.26 ^a,c^
GPx (nmol/min/mg protein)	5.60 ± 1.00 ^a^	3.79 ± 0.43 ^b^	4.04 ± 0.63 ^b^	4.16 ± 0.76 ^b^	4.03 ± 0.42 ^b^
Plasma					
MDA (µM)	31.1 ± 7.6 ^a^	66.7 ± 16.6 ^b^	58.3 ± 13.3 ^b,c^	45.3 ± 14.5 ^c^	47.3 ± 4.97 ^c^

All values are the mean ± SD (*n* = 8); ^a, b, c^ Values with different superscripts significantly differ between groups (*p* < 0.05); C, control group; D, diabetic group; L, low-dosage group (10 mg melatonin/kg of body weight (BW)); M, medium-dosage group (20 mg melatonin/kg of BW); and H, high-dosage group (50 mg melatonin/kg of BW).

**Table 7:** Effect of melatonin on plasma adipokines after the six-week melatonin intervention.

Group	C	D	L	M	H
Adiponectin (µg/mL)	3.98 ± 0.60 ^a^	2.14 ± 0.21 ^b^	2.82 ± 0.75 ^c^	2.79 ± 0.44 ^c^	2.90 ± 0.45 ^c^
Leptin (ng/mL)	5.98 ± 2.61 ^a^	4.94 ± 1.69 ^a^	3.96 ± 1.40 ^a,b^	2.08 ± 1.41 ^b^	2.41 ± 1.91 ^b^
leptin/adiponectin ratio	1.72 ± 0.92 ^a,b^	2.01 ± 0.65 ^a^	1.27 ± 0.24 ^b^	0.53 ± 0.32 ^c^	0.61 ± 0.68 ^c^
Melatonin (pg/mL)	60.4 ± 7.9 ^a^	50.3 ± 5.8 ^b^	120.5 ± 11.7 ^c^	148.6 ± 17.4 ^d^	134.8 ± 12.2 ^d^

All values are the mean ± SD (*n* = 8). ^a, b, c, d^ Values with different superscripts significantly differ between groups (*p* < 0.05). C, control group; D, diabetic group; L, low-dosage group (10 mg melatonin/kg of body weight (BW)); M, medium-dosage group (20 mg melatonin/kg of BW); and H, high-dosage group (50 mg melatonin/kg of BW).

**Table 8:** Pharmacokinetics of melatonin.

Group		10 mg/kg of BW	50 mg/kg of BW	*p* value
Melatonin level after oral administration of melatonin (µg/mL)				
10 min	Melatoninconcentration (μg/mL)	0.82 ± 0.10	3.62 ± 0.36	<0.01
30 min		1.82 ± 0.12	8.04 ± 0.59	<0.01
60 min		0.86 ± 0.02	3.84 ± 0.22	<0.01
120 min		0.17 ± 0.03	0.87 ± 0.09	<0.01
AUC (µg * min/mL)		102.0 ± 4.8	455.1 ± 25.2	<0.01
Ke (1/h)		1.62 ± 0.13	1.48 ± 0.15	0.126
Half-life (h)		0.43 ± 0.04	0.47 ± 0.05	0.122

* All values are the mean ± SD (*n* = 6); BW, body weight; AUC, area under the curve; and Ke, elimination rate constant.

## Abbreviations

ANOVA	an analysis of variance
BW	body weight
DM	diabetes mellitus
ELISA	enzyme-linked immunosorbent assay
FFA	fatty acid
FBG	fasting blood glucose
GPx	glutathione peroxidase
HOMA-IR	homeostasis model assessment for insulin resistance
IACUC	nstitutional Animal Care and Use Committee
MDA	Malondialdehyde
NA	nicotinamide
OGTT	Oral glucose tolerance test
ROS	reactive oxygen species
STZ	streptozotocin
SOD	superoxide dismutase
TBARS	thiobarbituric acid-reactive substance
ZDF	Zucker diabetic fatty
